# Gamma-Band Activities in Mouse Frontal and Visual Cortex Induced by Coherent Dot Motion

**DOI:** 10.1038/srep43780

**Published:** 2017-03-02

**Authors:** Hio-Been Han, Eunjin Hwang, Soohyun Lee, Min-Shik Kim, Jee Hyun Choi

**Affiliations:** 1 Department of Psychology, Yonsei University, Seoul, Republic of Korea; 2 Center for Neuroscience, Korea Institute of Science and Technology, Seoul, Republic of Korea; 3 Department of Neuroscience, University of Science and Technology, Daejeon, Republic of Korea

## Abstract

A key question within systems neuroscience is to understand how the brain encodes spatially and temporally distributed local features and binds these together into one perceptual representation. Previous works in animal and human have shown that changes in neural synchrony occur during the perceptual processing and these changes are distinguished by the emergence of gamma-band oscillations (GBO, 30–80 Hz, centered at 40 Hz). Here, we used the mouse electroencephalogram to investigate how different cortical areas play roles in perceptual processing by assessing their GBO patterns during the visual presentation of coherently/incoherently moving random-dot kinematogram and static dots display. Our results revealed that GBO in the visual cortex were strongly modulated by the moving dots regardless of the existence of a global dot coherence, whereas GBO in frontal cortex were modulated by coherence of the motion. Moreover, concurrent GBO across the multiple cortical area occur more frequently for coherently moving dots. Taken together, these findings of GBO in the mouse frontal and visual cortex are related to the perceptual binding of local features into a globally-coherent representation, suggesting the dynamic interplay across the local/distributed networks of GBO in the global processing of optic flow.

Perceptual binding, the process of integrating sensory information into a high-order coherent object representation (*e. g.*, visual attention, gestalt perception, and sensory awareness) has been reported to be accompanied by gamma-band oscillations (GBO, 30–80 Hz, central frequency at 40 Hz). For example, in human EEG, perception of flip-flopping gestalt figures as one image enhanced visual GBO^1^. Facial perception from individual face components induced GBO in posterior-central cortex^2^, which were diminished for inverted or unfamiliar faces^3^. GBO induced by the perceptual binding process have been reported to appear in the form of a distributed network for the more difficult visual object recognition tasks, such as maintaining the object representation in the visual working memory^4^ or identifying a hardly-recognizable scrambled stimuli^5^, or during perceptual grouping through contour integration^6^, which all often involve prefrontal/frontal GBO. Given that prefrontal/frontal GBO activities may involve top-down attentional selection^7^, frontal GBO were shown to advance visual GBO along with faster attentional latencies in monkey spike/LFP recordings, indicating that the long-range synchronization of GBO is associated with better perceptual processing for regulating communication across cortical components^8^. Additional evidence for the role of GBO in perceptual binding aligns with studies in clinical populations with fragmented perception such as schizophrenia^9^ or autism^10^,^11^,^12^.

Spatio-temporal binding of optic flow is one of the most fundamental functions of the visual system, which enables identification of the object by extracting temporally invariable rigid motion and defining configuration information from non-rigid chaotic optic flow in the visual scene. In laboratory experiments, the random-dot kinematogram (RDK) is one of the widely used paradigms for perceptual binding studies. RDK is typically composed of densely scattered dots in the screen, which move arbitrarily like Brownian motion or coherently for the observer to report the direction. By controlling the ratio of the coherently moving dots or the range of direction in RDK, the observer’s perception level for the global pattern can be determined. RDK requires the integration of local motion vectors partially intermingled with different directions of motion to identify dominant optic flow perceived to be globally coherent. Studies with RDK have also reported induced GBO in visual cortex, which support the idea of GBO as neural correlates of perceptual binding that integrates spatially and/or temporally separate sensory features into coherent representation^13^,^14^. Patients with GBO-related neuropsychiatric disorders also showed impaired performance on the RDK-based tasks^15^,^16^,^17^.

Previous studies have demonstrated that mice can discriminate the dominant direction of coherently moving components in RDK display^18^. Although few studies investigated electrophysiological responses to visually-presented RDK stimuli in early visual cortex from the anesthetized mouse brain^19^,^20^, relevant EEG studies focusing on the perceptual binding process in the mouse brain, comparable to human GBO studies, have not been documented.

In the current study, we investigated the visual and frontal GBO in relation to perceptual binding with RDK paradigm in mice. In human, the more coherent RDK induced the stronger GBO in visual cortex^13^. We used the same RDK patterns in awake mice, and compared the responses to three discrete conditions with static dots, randomly moving dots, and coherently moving dots. We first aim to observe the GBO activities in visual and frontal cortex for three different conditions of RDK. Secondly, the dynamic interplays of visual and frontal GBO in relation to RDK conditions are studied. We further developed a novel analysis tool, called *quantized spectrogram* to identify the period and frequency range of temporally unlocked GBO.

## Methods

All surgical and experimental procedures were followed by Korean Animal and Plant Quarantine Agency Publication No. 12512, partial amendment 2014, conforming to NIH guidelines (NIH Publication No. 86-23, revised 1985). All the procedures were approved by Korea Institute of Science and Technology and Institutional Animal Care and Use Committee of Korea Institute of Science and Technology (AP-2014L7002).

### Animals and surgery

Experiments were conducted on four C57BL/6 adult male mice. To implant the microscrew electrodes, surgical procedures were performed under deep anesthesia with ketamine (120 mg/kg, intraperitoneal) and xylazine (6 mg/kg, intraperitoneal). Once the subject was placed onto the stereotaxic apparatus, sterilized microscrew electrodes (Asia Bolt, South Korea) were fixed onto the skull surface of frontal (anteroposterior, 2.0 mm; mediolateral, ±2.0 mm) and visual cortex (anteroposterior, −3.75 mm; mediolateral, ±4.00 mm) bilaterally, with ground/reference electrodes on the occipital bone above the cerebellum. The electrode coordinates were determined according to the mouse atlas^21^ and are depicted in Supplementary Fig. S1. Dental cement (Vertex^TM^ Self-Curing, Vertex-Dental, Netherlands) was applied to secure the position of the electrodes. For the purpose of head-fixation during the experiment, one or two polycarbonate nuts (inner diameter 3 mm, Nippon Chemi-Con, Japan) were attached to the caudal edge of the cement. After surgery, mice were treated with antibiotics and analgesics.

### Experimental protocol

Mice were mildly water-restricted for 7–14 days before performing experiments (approximately 1 ml of water supplement per day). Health status was monitored according to a daily-basis health monitoring and systematic protocol for health status assessment (adopted from Guo *et al*.^22^). In the water restriction period, the mice were habituated to a head restraint for at least 15 min a day. During the recording session, the eyes were located to the horizontal plane with respect to the screen center. The visual angle (°) was the degree of arc from the two eyes’ midpoint to the visual stimuli on the screen.

Three conditions of visual stimuli were given: static dots (‘*Static*’), incoherent RDK (‘*Incoherent*’), and coherent RDK (‘*Coherent*’). All stimuli consisted of about fifty white dots (diameter 1.5°) scattered in black screen over a circular visual field with a diameter of 60°. In the ‘*Static*’ condition, randomly scattered dots were displayed. In the ‘*Incoherent*’ condition, each individual dot moved with the randomly chosen angle, and in the ‘*Coherent*’ condition, all the dots moved in one direction, which was randomly chosen in each trial. The speed of dot was 80 °/s and single motion lasted up to 800 ms. The duration of a stimulus was 4 s.

The stimuli were presented via MATLAB (MathWorks, Natick, MA, USA) with Psychophysics Toolbox^23^ on a 60 Hz TFT-LCD monitor (TRL-120WD, Tara LCD, South Korea), located 15 cm in front of the eyes of the animal. The dot-background contrast was 3.4, where the luminance of background and dots were 35 and 155 lux, respectively.

Each trial was composed of 0.5 s fixation and 4 s of RDK, and the inter-trial interval was randomly chosen between 5–8 s. During the fixation period, a white circle with diameter size of 3° was placed at the screen center. A total of 240 trials were performed, shuffling the order of the three conditions (80 trials per condition). To keep the mouse from falling into a sleep, a water droplet (0.04 ml) was released every 24 trials through a custom-built water delivery apparatus. When water was released, no stimulus was presented and a break of about 30 s was given before the following trial.

### Water delivery

During recordings, mice were provided with water through a water delivery apparatus to keep them from closing their eyes or falling into a sleep. Water was delivered through a polyethylene tubing (inner diameter 0.38 mm, Intramedic Corp., Becton, Dickinson and Company, NJ, USA) by a syringe pump (Fusion 100, Chemyx, Stafford, TX, USA). The licking position was set carefully in such a way that the animal was able to lick the water spout with no head movement. Normally, the tube end was located 2–4 mm below the mouth. Distilled water was supplied.

### EEG data acquisition and preprocessing

EEG data were collected in a Neuroscan SynAmps2 amplifier system (Compumedics, Charlotte, NC, USA) at a 2 kHz sampling rate. The impedance of each electrode was kept below 300 kΩ, and online filtering (60 Hz notch and 1–200 Hz band-pass) was applied. For processing, 10 s of epochs were extracted from the continuous data during a period that begins 5 s before the RDK stimulus onset. To align voltage fluctuation from trial to trial, a mean value of 500 ms period before fixation onset was subtracted from each epoch. Epochs containing artifacts were identified by amplitude threshold (absolute value larger than 450 *μ*V) or visual inspection and then were excluded from further analysis.

### Data analysis

The oscillatory activity during the RDK perception is considered to be induced oscillation because the oscillations are not evoked directly after the stimulus but emerged at a different time slot, possibly due to some distinct high-order process^24^,^25^. Given the trial-to-trial variability in the responses in terms of amplitude and latency, the power of induced oscillations is less likely to be ensured in averaging across trials. While induced oscillations are neither time nor phase locked to stimulus onset, the evoked oscillations are both time and phase locked to stimulus onset^26^,^27^. Here, we eliminated the evoked oscillation by subtracting the event-related potentials from the individual trial, then investigated the dynamics of induced oscillations.

#### Power spectrogram

A power spectrogram was obtained by applying fast Fourier transform with sliding Hanning window (512 ms) to the ERP-free trial epochs, which resulted in about 2 Hz spectral resolution. In each trial, the spectral power of each frequency component was normalized by subtracting the mean baseline (0.5–4 s before the stimulus onset) power, and then averaged across trials.

#### Quantized spectrogram

As induced GBO can be characterized by the increase of the band power, we quantized the power spectrogram to capture the moment at which oscillatory EEG activity occurs by assigning 1 for the significantly enhanced power and 0 for the rest at a specific frequency and time. We referred to the digitized spectrogram as a *quantized spectrogram* (QS). The QS is a two-dimensional matrix represented in time and frequency domain. The advantage of adopting the QS matrix is radically simple and recapitulates the complex responses without blurring individual activities. Based on the QS matrix, we investigated the events GBO power increased, and their relationship across the brain regions.

The QS matrix was defined by the following steps: First, the confidence interval of baseline power was calculated within 1 standard deviation, which corresponds to 68%^28^. The power values of the baseline period were used as a sample for comparison. Secondly, the QS element at a specific frequency and time, 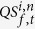 was determined to be 1 if the correspondent power exceeds the confidence interval or 0 otherwise. That is,


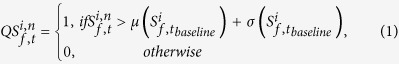


where 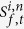 is the power of EEG in the *n*-th trial in the *i*-th channel at time, *t* and frequency, *f*. The size of time and frequency bins were 0.5 ms and 1.95 Hz, respectively, with a sliding time window of 512 ms.

#### Probability matrix

The probability matrix, 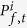 was obtained to estimate the occurrence probability of GBO on each channel, by averaging 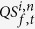 over trials and dividing by the total number of trials, *N*. 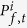 ranges from 0 (absence of trials with enhanced GBO) to 1 (enhanced GBO observed for all the trials) and is defined by:


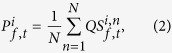


#### Joint probability matrix

**Figure 1:**
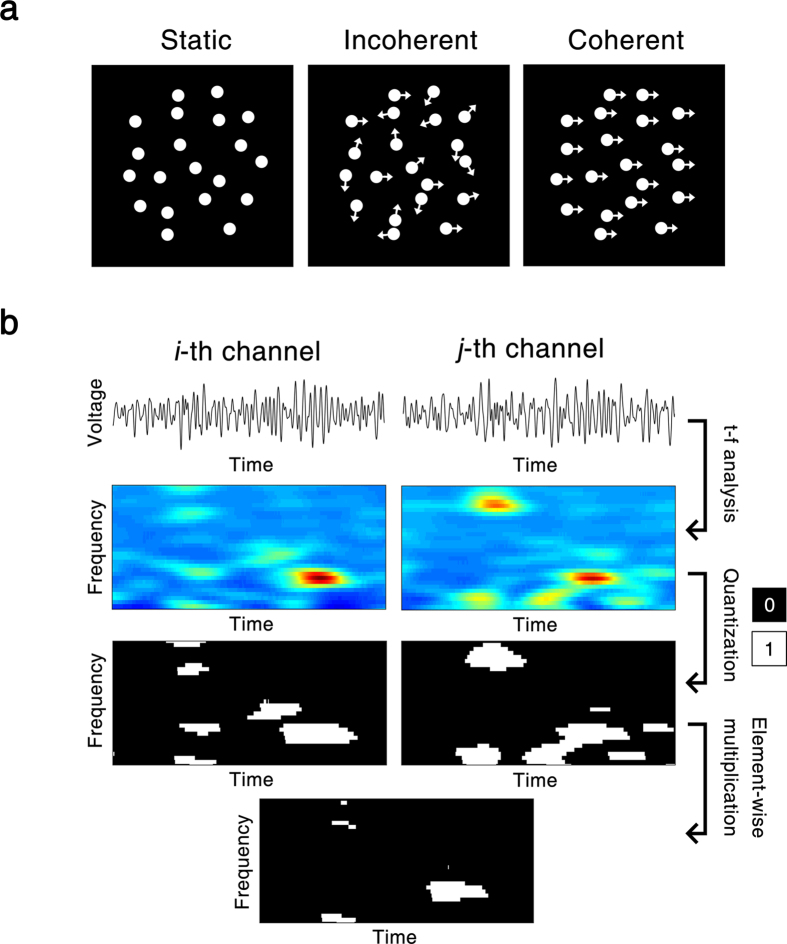
Schematic illustrations showing (**a**) visual stimuli of RDK and (**b**) data processing sequence for obtaining quantization matrix.

To capture the concurrent increase of oscillatory power over two channels (*i. e.*, simultaneous enhancement of power at a specific frequency), a joint probability matrix, 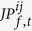 was calculated. First, an entry-wise product between channel QS matrices was calculated. Next, the product was summated over the trials and divided by *N* as below:


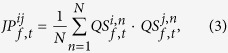


where 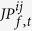 is the joint probability of *i*-th and *j*-th channels. This joint probability was used to scale the functional connectivity between all channels as well. The current event of enhancement of GBO across all the channels was referred to as the all-channel joint probability matrix, 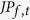 defined by


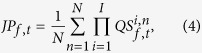


where *I* is the total number of channels. The analysis scheme using QS is depicted in Fig. 1b.

#### Statistical tests

Wilcoxon t-tests were performed to identify whether the mean value of gamma band activities during the perception of RDK (0.5–4 s after the stimulus onset) is statistically greater than that of the baseline period (0.5–4 s before the stimulus onset). The frequency band was chosen between 30–50 Hz for statistical tests, as preliminary analysis revealed that induced activities in response to the visual stimuli were particularly prominent in this band (see Supplementary Figs S2–3). To reduce the chance of obtaining type I errors, p-values in the Wilcoxon t-tests were corrected by multiplying the number of tests. In addition, to compare the extent of effects between RDK display conditions, non-parametric versions of ANOVA (analysis of variance; Kruskal-Wallis test) and *post hoc* tests with Tukey method were performed. Test statistics, z and H were calculated from the sum of signed ranks. The alpha level for all the tests described above was 0.05, and the t-tests were one-sided.

## Results

After rejection of artifacts, a total of 895 trial epochs out of 960 (93.2%) were included in the analysis; 306 for ‘*Static*’ (95.6%), 291 for ‘*Incoherent*’ (90.9%), and 298 for ‘*Coherent*’ (93.1%). As no hemispheric difference was noticeable in a preliminary analysis, left and right EEG activities were averaged after calculating time-frequency analysis in each area, the frontal and the visual.

### GBO induced by RDK

**Figure 2:**
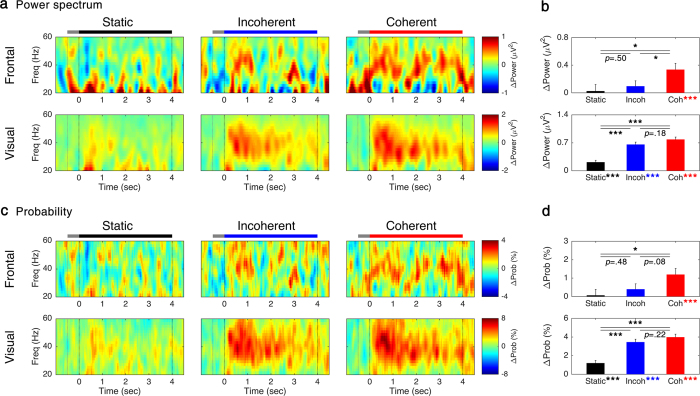
Time-frequency maps of GBO. Time-frequency representation of grand-averaged (**a**) power spectrum and (**c**) probability of GBO occurrence for each condition at frontal (upper row) and visual cortex (lower row) and (**b,d**) average of 30–50 Hz frequency components within 0.5–4 s. The black, blue, and red lines indicate the period of stimulus presentation, and gray lines indicate fixation period. Error bars represent SEM. *p < 0.05; **p < 0.01; ***p < 0.001.

Figure 2a illustrates grand-averaged spectral power of frontal and visual EEG, showing more pronounced responses in the visual cortex. In the frontal cortex, a significant increase of the GBO power was observed for the ‘*Coherent*’ condition (*z* = 3.488, *p* < 0.001), but not for the other conditions (*z* = 0.525, *p* = 0.899. for ‘*Static*’; *z* = 0.692, *p* = 0.734. for ‘*Incoherent*’). ANOVA and *post hoc* multiple comparisons revealed that the power difference across the display conditions was statistically significant (*H*(2) = 6.201, *p* < 0.05 and was the strongest at ‘*Coherent*’, *p* < 0.05 for ‘*Coherent*’ *versus* ‘*Static*’; *p* < 0.05 for ‘*Coherent*’ *versus* ‘*Incoherent*’). There was no statistically significant difference between ‘*Static*’ and ‘*Incoherent*’ for frontal GBO power (*p* = 0.498).

In the visual cortex, the GBO power was statistically significantly higher than the baseline power (*z* > 4.5, *p* < 0.001 for all the conditions). The ANOVA for the average GBO power in the visual cortex revealed that the difference across the display conditions were statistically significant (*H*(2) = 47.208, *p* < 0.001). Unlike for the frontal area, *post hoc* multiple comparisons showed no statistically significant difference between ‘*Coherent*’ *versus* ‘*Incoherent*’ (*p* = 0.182). The moving dots showed a stronger power than static dots (*p* < 0.001 for ‘*Incoherent*’ *versus* ‘*Static*’; *p* < 0.001 for ‘*Incoherent’ versus* ‘*Static*’). These results imply a distinctive role for the perceptual processing between frontal and visual GBO, particularly showing the different preference for coherence of the motion and of the motion itself. The results of statistical tests are shown in Fig. 2b.

The transient increase of oscillatory power was converted to the QS matrix in each trial to log the event time by time and frequency by frequency. After that, a probability matrix was calculated over the QS matrices. Generally, the spectrogram patterns of the probability matrix follow the patterns of power as depicted in Fig. 2c. As observed for the GBO power spectrum, a more prominent occurrence of enhanced GBO events was observed in the visual cortex compared to the frontal cortex. A close examination showed that noticeable changes in the low-frequency power (near 20 Hz) are invisible in the spectrogram of the probability matrix. Statistical tests on the probability matrix over display conditions led to the same results with statistical tests on the GBO powers: In the frontal cortex, the probability for enhanced GBO events increased in ‘*Coherent*’ (*z* = 3.404, *p* < 0.001), but did not increase under other conditions (*z* = 0.576, *p* = 0.848 for ‘*Static*’; *z* = 1.112, *p* = 0.400 for ‘*Incoherent*’). The main effect of the display condition was marginally significant (*H*(2) = 5.294, *p* = 0.071). In the visual cortex, the probability of enhanced GBO events was statistically significantly higher in all the conditions compared to baseline GBO (*z* > 4.6, *p* < 0.001 for all the conditions; *H*(2) = 42.877, *p* < 0.001). In the visual cortex, there was no statistically significant difference between ‘*Coherent*’ and ‘*Incoherent*’ (*p* = 0.218). *Post hoc* comparisons for other conditions revealed the lowest probabilities of occurrence of increased GBO events in ‘*Static*’ (*p* < 0.001 for ‘*Static*’ *versus* ‘*Incoherent*’; *p* < 0.001 for ‘*Static*’ *versus* ‘*Coherent’*). The results of statistical tests are shown in Fig. 2d.

### Functional connectivity of GBO induced by RDK

**Figure 3:**
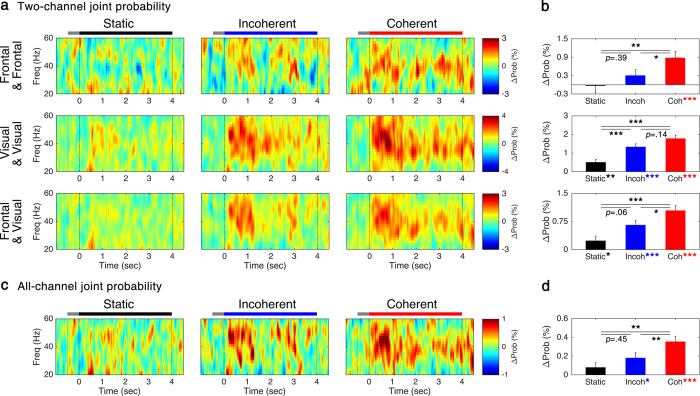
Joint probability matrix. (**a**) Two-channel joint probability matrix of each pair of electrodes and (**c**) all-channel joint probability matrix and (**b,d**) its average of 30–50 Hz components within 0.5–4 s, respectively. The black, blue, and red lines indicate the period of stimulus presentation, and gray lines indicate fixation period. Error bars represent SEM. *p < 0.05; **p < 0.01; ***p < 0.001.

Since the induced oscillations are neither time- nor phase-locked, it is difficult to apply conventional measure to scale the functional influences of induced GBO. Under the assumption that co-occurred GBO are functionally related, we evaluated the joint probability matrix and compared for different RDK conditions. By definition, the joint probability matrix indicates the occurrence rate of concurrent GBO in different brain regions. The upper and middle panels in Fig. 3a show the connectivity within the frontal and visual area, respectively. The lower panel in Fig. 3a shows the connectivity between frontal and visual area. The joint probability matrices for frontal-visual pairs were averaged over four possible channel combinations (*i. e.*, left/right, frontal/visual channels) as preliminary analysis revealed no difference was noticeable between them. The results of statistical tests are summarized in Fig. 3B. It is noticeable that all the stimuli increased connectivity within the visual area compared to baseline values (*z*s > 3.3, *p*s < 0.01 for all the conditions). On the other hand, only coherently moving dots increased the connectivity within the frontal area compared to baseline value (*z* = 0.088, *p* = 1.395 for ‘*Static*’; *z* = 1.140, *p* = 0.381 for ‘*Incoherent*’; *z* = 4.102, *p* < 0.001 for ‘*Coherent*’). In the case of frontal-visual connectivity, all the stimuli increased the connectivity values (*z* = 2.547, *p* < 0.05 for ‘*Static*’; *z* = 4.918, *p* < 0.001 for ‘*Incoherent*’; *z* = 7.120, *p* < 0.001 for ‘*Coherent*’).

In *post hoc* comparisons between ‘*Static*’ *versus* ‘*Coherent*’, the connectivity increased in all the pairs (*p*s < 0.01 for all conditions). However, in comparisons of ‘*Static*’ *versus* ‘*Incoherent*’, the connectivity between pairs showed different results (*p* < 0.001 for visual pair, *p* = 0.391 for frontal pair, *p* = 0.060 for frontal-visual pairs). With respect to ‘*Incoherent*’ *versus* ‘*Coherent*’, only the visual pair did not show any statistically significant difference (*p* = 0.141 for visual pair; *p* < 0.05 for frontal pair; *p* < 0.05 for frontal-visual pairs). The results of *post hoc* comparisons are depicted in Fig. 3b.

The joint probability across all the channels was also calculated in order to estimate the synchrony level of the global network and it is shown for different conditions in Fig. 3c. RDK increased the global synchrony for moving dots (*z* = 2.264, *p* < 0.05 for ‘*Incoherent*’; *z* = 6.045, *p* < 0.001 for ‘*Coherent*’), but the increase was marginal for static dots (*z* = 1.997, *p* = 0.069). ANOVA revealed the differences between display conditions (*H*(2) = 12.789, *p* < 0.01). *Post hoc* comparisons showed coherent RDK produced a greater response than others (*p* < 0.01 for ‘*Coherent’ versus* ‘*Incoherent*’; *p* < 0.01 for ‘*Coherent’ versus* ‘*Static*’), while there was no statistically significant difference between incoherent RDK and static dots (*p* = 0.447).

## Discussion

In this study, we investigated GBO activities in the mouse frontal and visual cortex modulated by passive perception of various conditions of dot display. While GBO in the visual cortex were enhanced by viewing coherently and incoherently moving dots, GBO in the frontal cortex were modulated only by coherently moving dots. GBO have been implicated in various cortical processes of perception, such as binding of distributed representations^29^,^30^ or attentional modulation of sensory signals^31^,^32^,^33^. By far, the largest proportion of studies has been focused on the primary sensory cortex in human, cat, and monkey (for review see Engel & Singer^34^). Relatively few studies have been performed in rodents. As GBO have been widely accepted as a marker for neural synchrony for temporal binding of distributed information, the GBO activity patterns with respect to different visual features also need to be characterized in mice. The advantage of the RDK paradigm is that it differentiates the visual features in terms of static *versus* dynamic, and dynamic random-dot pattern *versus* strongly coherent dot motion. As previously reported in human^13^ and in mouse^19^, we observed that GBO are generated in visual cortex by seeing random dots, but the GBO were not particularly stronger for coherent dots as reported in human magnetoencephalogram study. To the best of our knowledge, frontal GBO activity during RDK has not been reported in mice. Nevertheless, the relevance of GBO activities in the frontal cortex for perception of random dots is predicted from the human studies^14^,^15^. Although the brain region is not exactly matching, in human EEG, GBO were significantly present in the central region for coherent dots, but not for incoherent dots^15^. This dissociation between visual and frontal cortex in terms of GBO activity might reflect a region-specific functional difference. For example, in processing the sensory information, a variety of non-sensory cognitive processes are activated and are specifically large for multistable visual inputs^35^ or at the moment of perceptual discovery of a global pattern^36^ or perceptual decision making^37^. Taken together, early and late processing of sensory inputs are represented in different brain networks, and our observation analyzes functionally distinctive contributions of visual and frontal cortex in processing different features of visual information in mice.

Numerous studies have addressed the characterization of GBO during perceptual processing in a variety of species, but a study in mouse model is relatively rare. In recent years, many new technologies have been developed for the dissection of neural circuits and most of them can be exclusively applied to mouse. For example, GBO studies of psychiatric disease models enable us to associate the functional roles of neuronal oscillations with the expression of behavioural symptoms^38^,^39^,^40^. Even though the functional relationship with high-order perceptual processing has been relatively well documented in the human brain, the document on the GBO representation for perceptual binding is lacking on mice. Without a doubt, there are gaps between human and mouse studies. Moreover, the induced GBO studies in human largely rely on complex visual stimuli (*e. g.*, the Kanizsa illusory triangle, the human face, and line-drawings of a fragmented object) that cannot be easily applied to mice. Nonetheless, the universal mechanisms underlying neuronal processing for perceptual binding are expected to be preserved.

Lastly, we analyzed occurrence of GBO through the QS matrix operation. This method of analysis allowed us not only to focus on the ongoing oscillatory events while leaving aside the unnecessary power-decreased oscillatory activities, but also to capture the concurrent oscillatory events across the channels as a simple probability representation. Furthermore, our observations with the joint probability matrix showed that the functional structures of brain regions in relation to enhanced GBO are modulated by different RDK display. This joint probability matrix is suitable for exploring the aberrance of the co-occurrence of enhanced oscillations in different brain regions. Being different from the phase synchrony based methods, the joint probability matrix can estimate the relationship between asynchronous oscillations. It is crucial to investigate the relative changes in the values for different tasks and to randomize the task sequence, as the brain state (*e. g.*, sleep or alertness) also leads the concurrent changes in power. Moreover, the GBO in different brain regions could be enhanced by same or different drivers. Hence, further investigations like physical perturbation (*e. g.*, optogenetic inhibition) or adaptation of mutual information index to QS may reveal the precise architecture of GBO. Notwithstanding, QS can be easily adapted to the development of an analysis framework to characterize oscillatory activities in multiple brain regions, reducing the complexity of multi-dimensional neuroimaging data, and easily extended to characterize the global or local functional changes in GBO related network.

Application of RDK to mouse model demonstrates that, as for human, distinct functional roles of frontal and visual cortex exist in the mouse model. In addition, quantizing the spectrogram reduces the complexity of the data analysis by focusing on the presence of induced GBO rather than their strength, and presents an elegant presentation of temporally unlocked GBO. Taking advantage of the mouse model, optogenetic interrogation or genetic perturbation may provide an opportunity to identify the functional anatomy corresponding to individual cortical processing, which is the fundamental building block for our understanding of perceptual binding. However, without expression of behaviour, perception of the animal is not recognized in the passive paradigm. Our results, compared to those of human studies, with relatively small differences between coherent versus incoherent RDK in terms of GBO pattern may arise from this problem^14^,^15^. Besides this, the absence of coherent gradation was limited to deliver any correlation between GBO intensity and coherence strength systematically as found by Siegel *et al*.^13^. Adaptation of rigorous psychophysical procedures is required to investigate the cortical process step by step (for review see Carandini & Churchland^41^). In a measurement point of view, the dissection of cortical circuits in terms of functional role will be improved by mapping the entire cortex (*e. g.*, high-density EEG^42^). A whole brain mapping with direct intervention in the processing of the sensory input will reveal the brain structure associated with multiple steps of perceptual binding.

## Additional Information

**How to cite this article**: Han, H.-B. *et al*. Gamma-Band Activities in Mouse Frontal and Visual Cortex Induced by Coherent Dot Motion. *Sci. Rep.* **7**, 43780; doi: 10.1038/srep43780 (2017).

**Publisher's note:** Springer Nature remains neutral with regard to jurisdictional claims in published maps and institutional affiliations.

## Supplementary Material

Supplementary Materials
