# Supplementary material for: Gamma-Band Activities in Mouse Frontal and Visual Cortex Induced by Coherent Dot Motion
Source: Sci Rep. 2017 Mar 2;7:43780. doi: 10.1038/srep43780 (PMC5333145; doi:10.1038/srep43780)
Supplement: Supplementary Materials [file srep43780-s1.doc]

**Supplementary Information Materials**

- Supplementary Figure S1
- Supplementary Figure S2
- Supplementary Figure S3

**Original Manuscript Information**

**Title:** Gamma-Band Activities in Mouse Frontal and Visual Cortex Induced by Coherent Dot Motion

**Author names:** Hio-Been Han^1,2^, Eunjin Hwang^2^, Soohyun Lee^2^, Min-Shik Kim^1^, Jee Hyun Choi^2,3, *^

**Affiliations:**^1^ Department of Psychology, Yonsei University, Seoul, Republic of Korea
^2^ Center for Neuroscience, Korea Institute of Science and Technology, Seoul, Republic of Korea
^3^ Department of Neuroscience, University of Science and Technology, Daejeon, Republic of Korea
^*^ Corresponding author

**Corresponding Author:** Jee Hyun Choi
Center for Neuroscience Korea Institute of Science and Technology 14-gil 5 Hwarang-ro, Seongbuk-gu, Seoul, 02792, Republic of Korea Phone: +82 (2) 958-6952 Fax: +82 (2) 958-6937 [jeechoi@kist.re.kr](mailto:jeechoi@kist.re.kr)


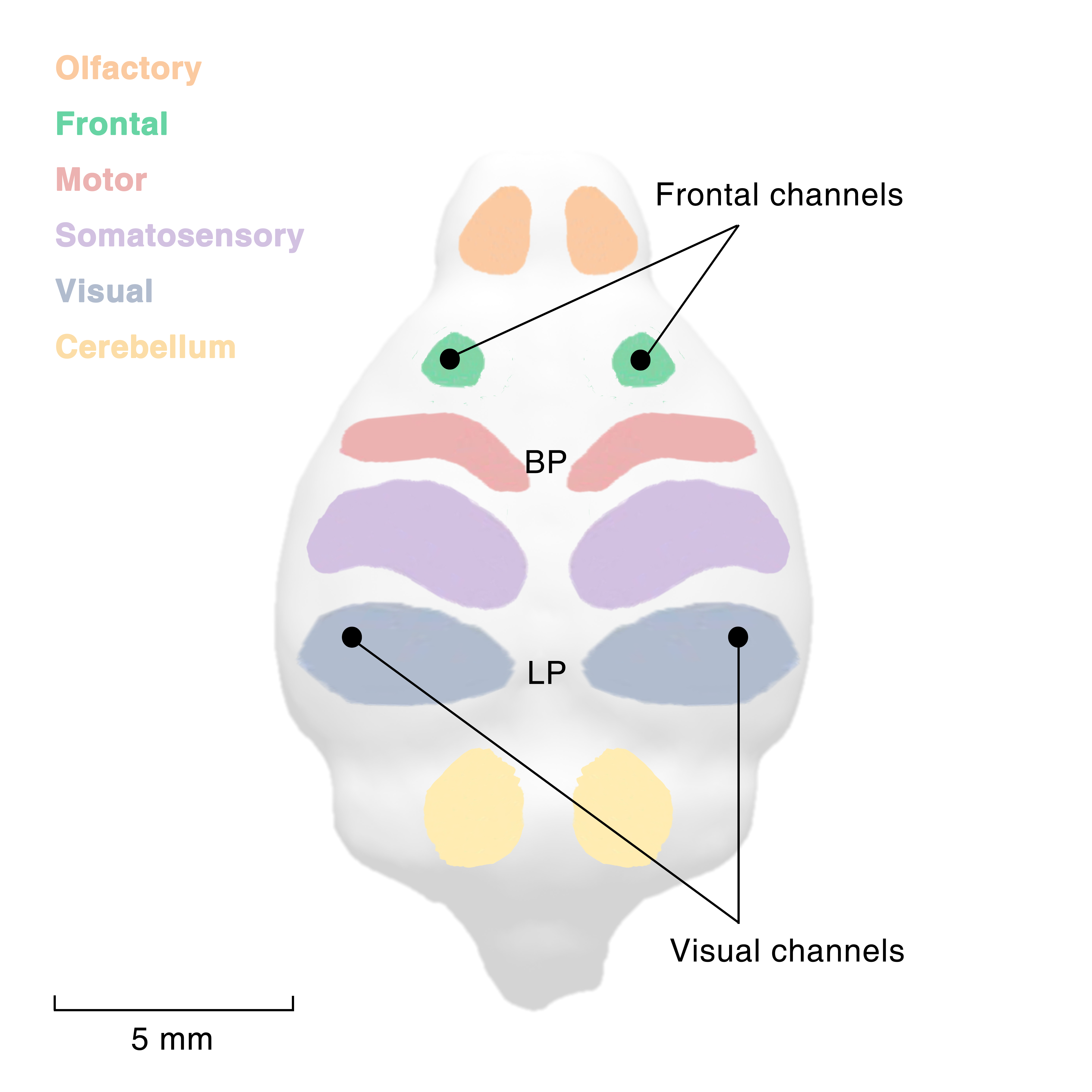


Supplementary Figure S1. Schematic illustration of channel location on model mouse brain. Channel positions are marked as filled black circle. Model brain template was rendered on Curry 7 software (Neuroscan Inc., Herndon, VA) using *in vitro* magnetic resonance image (MRI) data obtained from open database of the Magnetic Resonance Microimaging Neurological Atlas (http://brainatlas.mbi.ufl.edu/Database)^1^.


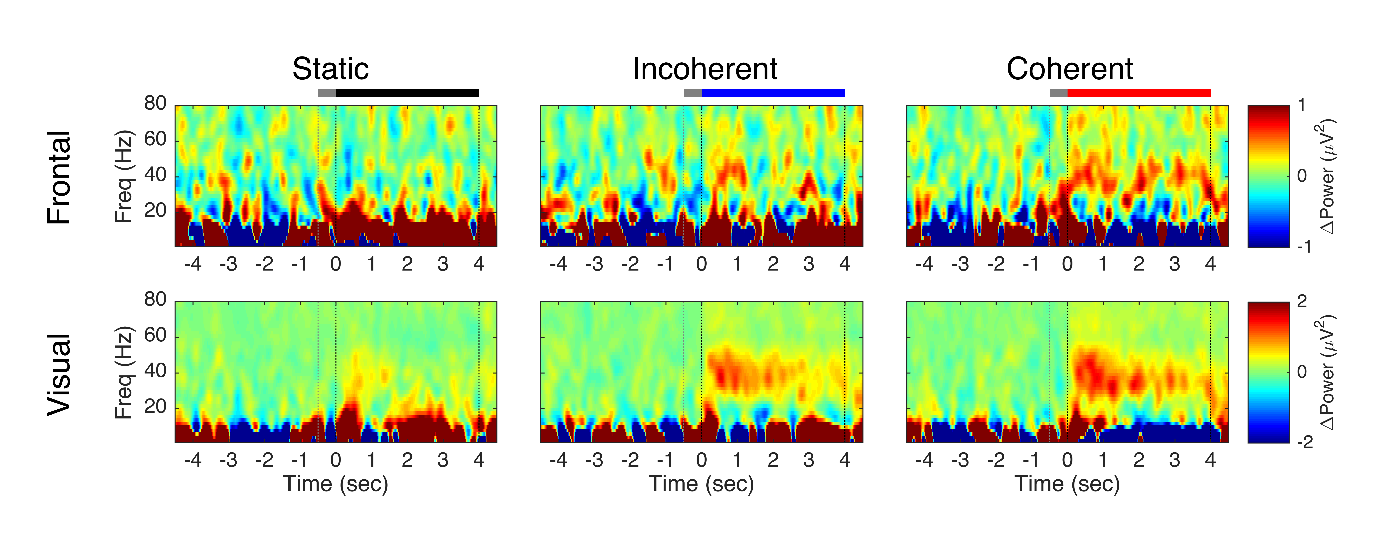


Supplementary Figure S2. Time-frequency maps of oscillatory power with broader time/frequency range. Grand-average power spectrum of 1–80 Hz activities for each condition at frontal (upper row) and visual area (lower row). The black, blue, and red lines indicate the period of stimulus presentation, and gray lines indicate fixation period.


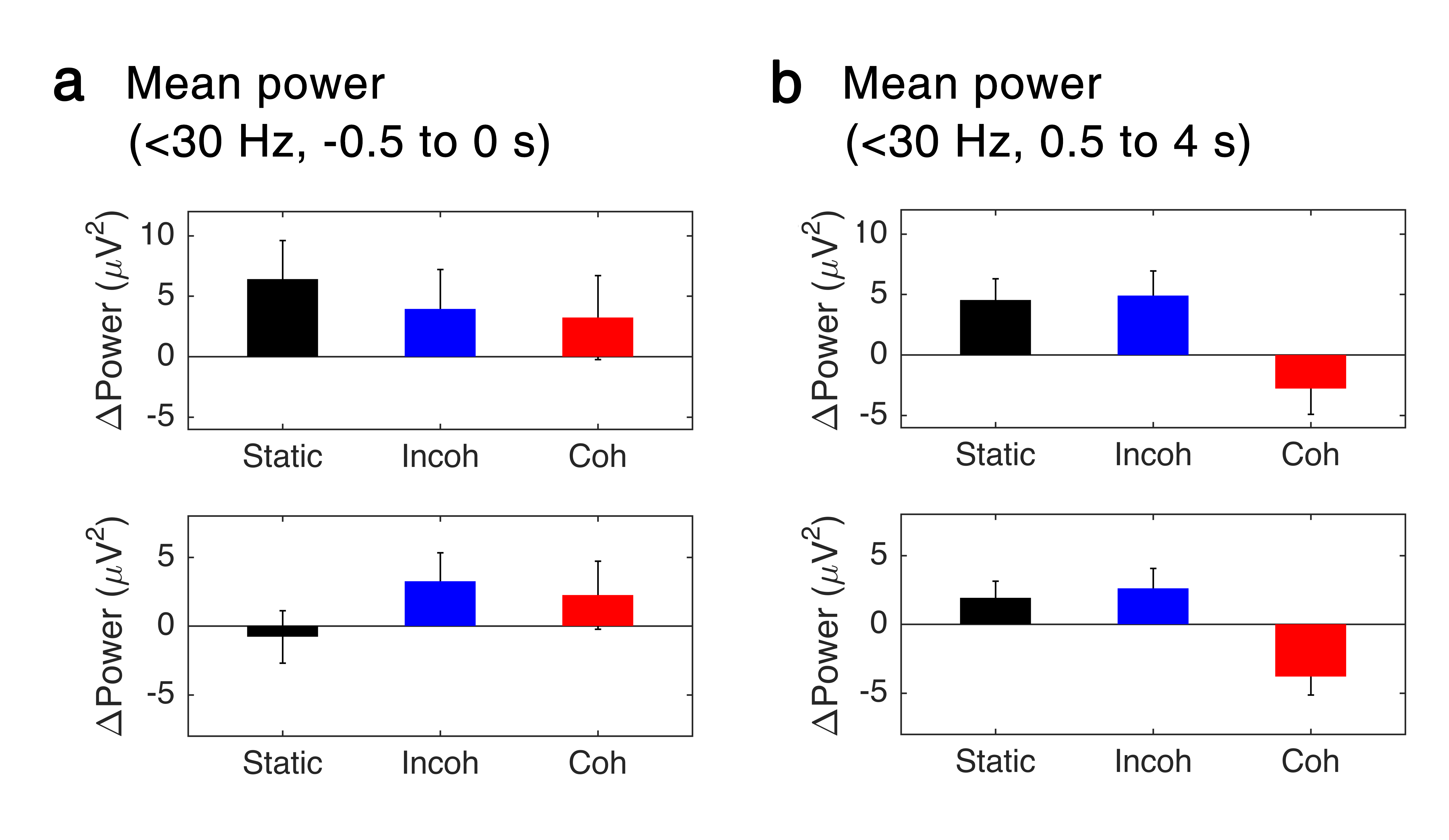


Supplementary Figure S3. Power analysis of low-frequency oscillations. Grand-average of power change of lower frequency band (<30 Hz). The power change was calculated by subtracting the mean power of baseline period from the mean power of fixation (a) or stimulus period (b). Non-parametric ANOVA revealed the main effect of display condition was non-significant on fixation period, *H*(2) = 1.088, *p* = .580 for frontal, *H*(2) = 2.025, *p* = .363 for visual. In stimulus period, the main effect of display condition was non-significant on frontal area, *H*(2) = 1.727, *p* = .422, but was significant on visual area, *H*(2) = 8.873, *p* < 0.01. The error bars represent SEM.

**References**

1. Ma, Y. *et al*. A three-dimensional digital atlas database of the adult C57BL/6J mouse brain by magnetic resonance microscopy. *Neuroscience* **135**(4), 1203-15 (2005)
